# Three-dimensional progressive correction of Madelung deformity using the Ilizarov technique in patients aged ≥12 years: a retrospective study

**DOI:** 10.3389/fped.2026.1767337

**Published:** 2026-03-26

**Authors:** Xinjian Pei, Kaixuan Tian, Wenjun Li, Xiaofei Yu, Yadong Yu

**Affiliations:** 1 Department of Pediatric Orthopedics, The Third Hospital of Hebei Medical University, Shijiazhuang, China; 2 Department of Hand Surgery, Beijing Jishuitan Hospital, Beijing, China; 3 Department of Hand Surgery, The Third Hospital of Hebei Medical University, Shijiazhuang, China

**Keywords:** Madelung deformity, Ilizarov technique, circular external fixator, three-dimensional correction, radial osteotomy, wrist joint function

## Abstract

**Background:**

Madelung deformity is a rare developmental wrist abnormality that often leads to joint pain and limited mobility. Traditional surgical methods typically struggle to simultaneously correct the abnormal bone angle and the associated bone shortening. This study assesses the efficacy and safety of the Ilizarov progressive three-dimensional orthopedic technique in treating Madelung deformity in patients aged 12 years or older.

**Methods:**

We performed a retrospective analysis of data from 19 patients aged 12 years or older with Madelung deformity who were treated with the Ilizarov technique between 2020 and 2024. This single-center study was a retrospective case series (Level IV evidence). During the surgery, we executed an osteotomy on the radius and installed an adjustable external fixator. Postoperatively, we made gradual adjustments to correct the wrist deformity angle and simultaneously lengthen the bone. We evaluated surgical outcomes with imaging measurements and functional scores and recorded all complications.

**Results:**

The study observed a significant improvement in both the angular deformity of the wrist and bone length among all patients. Additionally, there was a notable increase in the range of motion in the wrist joint and hand grip strength. Postoperative functional scores were excellent or good in 89.5% of cases. Only a few patients experienced mild pin tract infections, and no serious complications were reported.

**Conclusions:**

The Ilizarov technique successfully corrected angular and length abnormalities associated with Madelung deformity, significantly enhancing wrist function. It proved to be safe and can be recommended as a treatment option for individuals with moderate to severe deformity, particularly those with closing or closed physes. Further long-term follow-up studies are needed to confirm its lasting effects.

## 1 Introduction

Madelung deformity is a rare wrist disorder characterized by developmental abnormalities and inherited in an autosomal dominant pattern. It typically presents in late childhood or adolescence (1, 2). The primary pathological feature is a growth disturbance of the volar and ulnar epiphysis at the distal radius, causing the radiocarpal joint surface to incline towards the volar and ulnar sides. This often leads to dorsal subluxation of the distal ulna, resulting in wrist pain, restricted motion, and functional impairment (3). The Vickers ligament, which connects the lunate to the radius, is thought to exacerbate the deformity by restricting volar growth of the radius (4). The deformity accounts for about 1.7% of hand deformities and predominantly affects females, with a male-to-female ratio of approximately 1:4. It is frequently bilateral and may occur in isolation or with genetic syndromes such as Léri-Weill dyschondrosteosis and Turner syndrome, both associated with SHOX gene haploinsufficiency (2, 5).

Various surgical options are available for treating Madelung deformity. Traditional methods, such as distal radial wedge osteotomy with internal fixation, can correct angular abnormalities in the sagittal and coronal planes but often fail to address radial shortening and secondary ulnocarpal impaction (6–9). The Ilizarov circular external fixator technique presents an innovative solution, enabling progressive bone lengthening and three-dimensional adjustment to correct complex deformities (10–12). Given the limitations of traditional surgeries in addressing radial shortening and preventing ulnocarpal impaction, this study aims to systematically evaluate the efficacy and safety of the Ilizarov external fixator technique for the three-dimensional correction of Madelung deformity in patients aged 12 years or older.

## 2 Materials and methods

### 2.1 General information

This study retrospectively analyzed 19 patients with Madelung deformity treated using the Ilizarov technique from January 2020 to December 2024. It is a single-center retrospective case series (Level IV evidence). Inclusion criteria were: (1) imaging-confirmed Madelung deformity, defined by an ulnar inclination angle of the radius greater than 30° and a volar tilt angle exceeding 20°; (2) clinically significant wrist pain or functional impairment. Exclusion criteria were: (1) pseudomadelung deformity secondary to trauma or other conditions; (2) a history of severe osteoporosis or local infection; (3) incomplete clinical or imaging follow-up data; or (4) loss to follow-up.

The study group included 4 males and 15 females, with a mean age of 13.5 ± 1.2 years (range 12–17 years). At the time of surgery, 7 patients had open physes and 12 had closing or closed physes, so the cohort primarily comprised adolescents with near-mature or mature skeletons. Three patients had associated syndromes: two with Léri-Weill dyschondrosteosis and one with Turner syndrome. Bilateral deformity occurred in 11 patients; to avoid statistical clustering, only the more symptomatic side was analyzed for those cases. The hospital ethics committee approved the study, and informed consent was obtained from all patients' guardians.

### 2.2 Surgical techniques

#### 2.2.1 Preoperative planning

**Figure 1:**
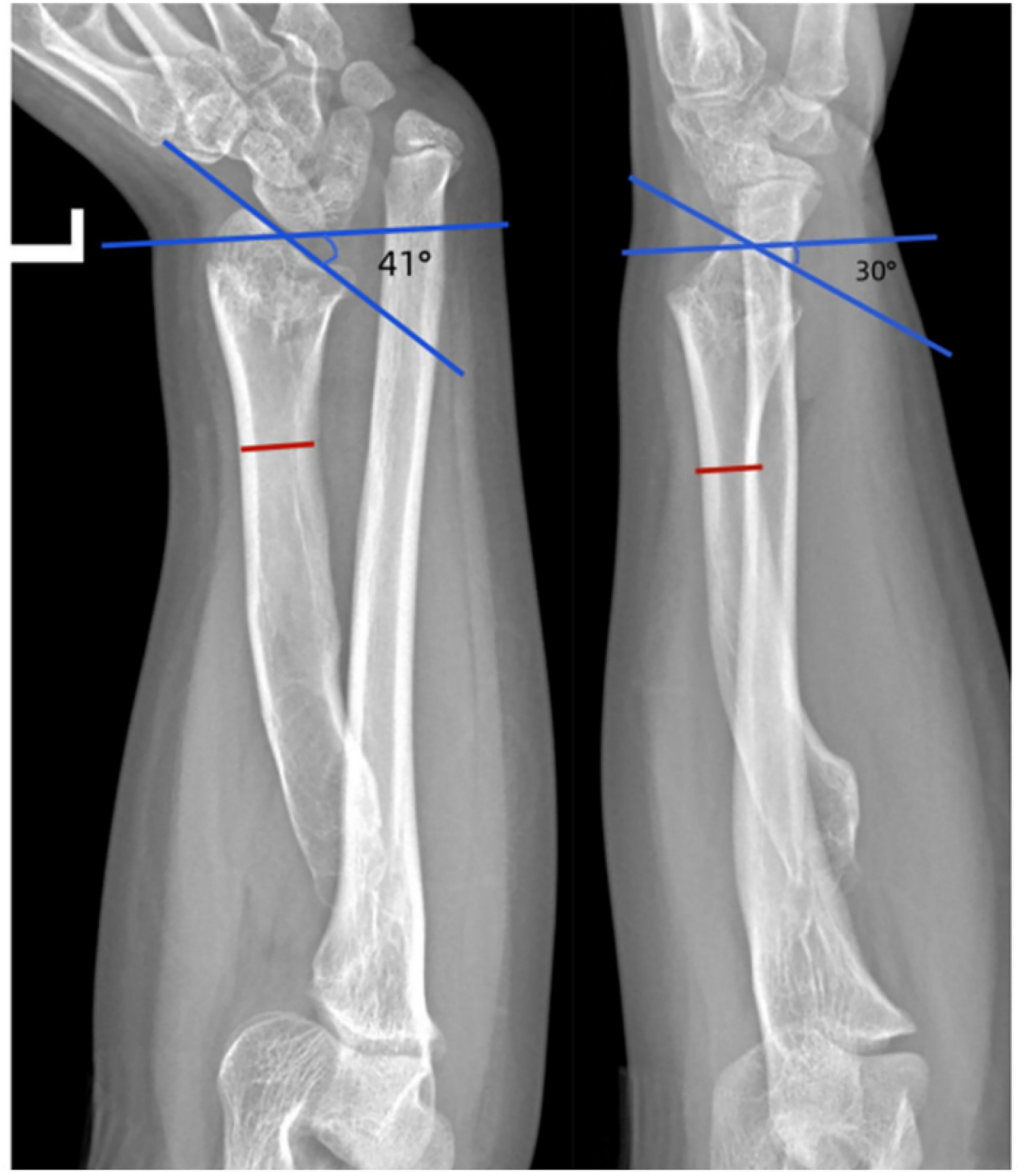
Anteroposterior and lateral radiographs demonstrating Madelung deformity. The blue-marked angles indicate the ulnar inclination and the volar tilt. The red line denotes the osteotomy plane.

We utilized standard anteroposterior and lateral x-ray images of the wrist joint (Figure 1) to measure the ulnar inclination and volar tilt angles. These angles are defined by the intersection of a line parallel to the joint surface with a line perpendicular to the longitudinal axis of the radius. The osteotomy plane was established 5–8 cm proximal to the metaphyseal region of the distal radius.

#### 2.2.2 Installation of external fixator and osteotomy

**Figure 2:**
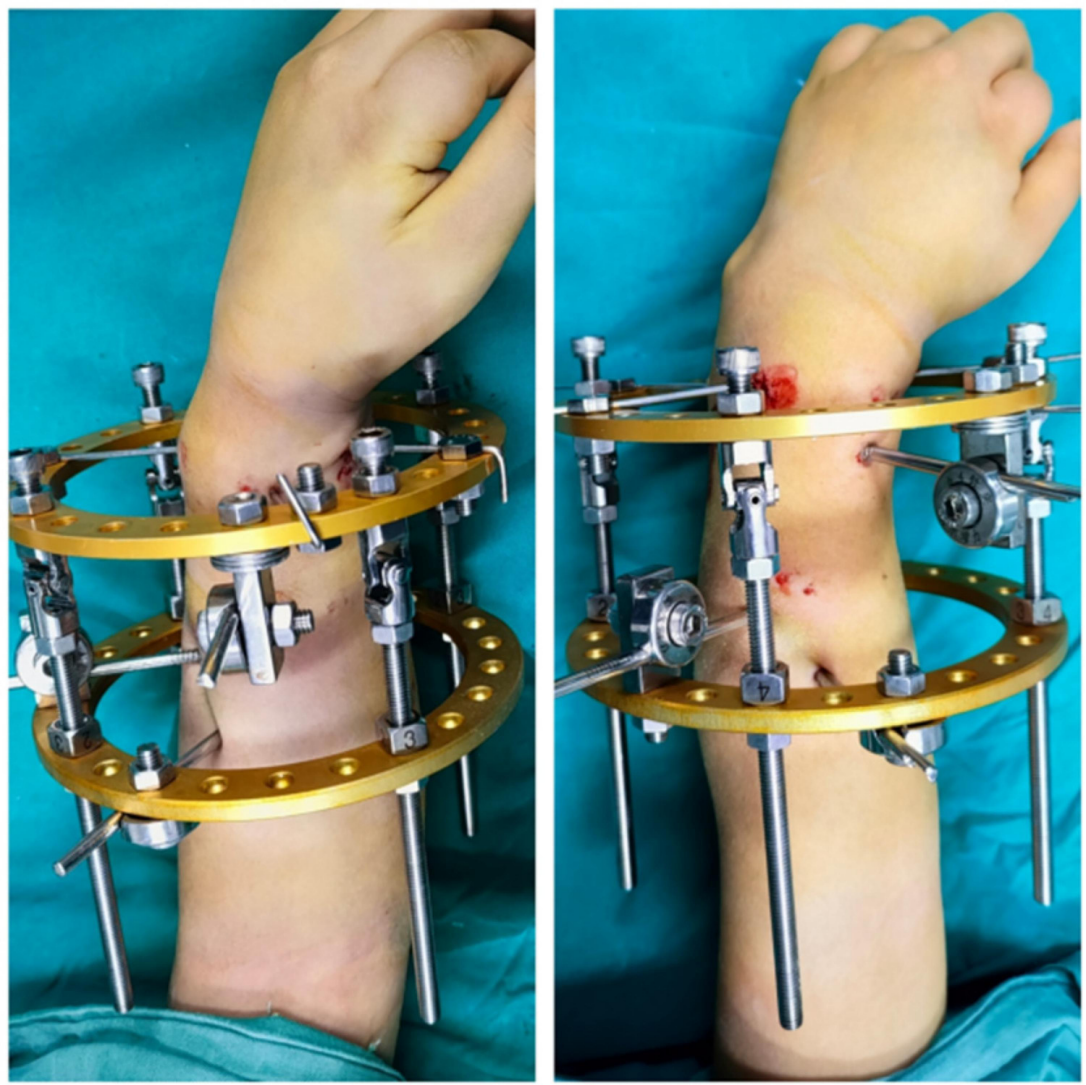
Assembly of the circular external fixator. The device is configured with crossed K-wires and extension rods in both the coronal and sagittal planes.

**Figure 3:**
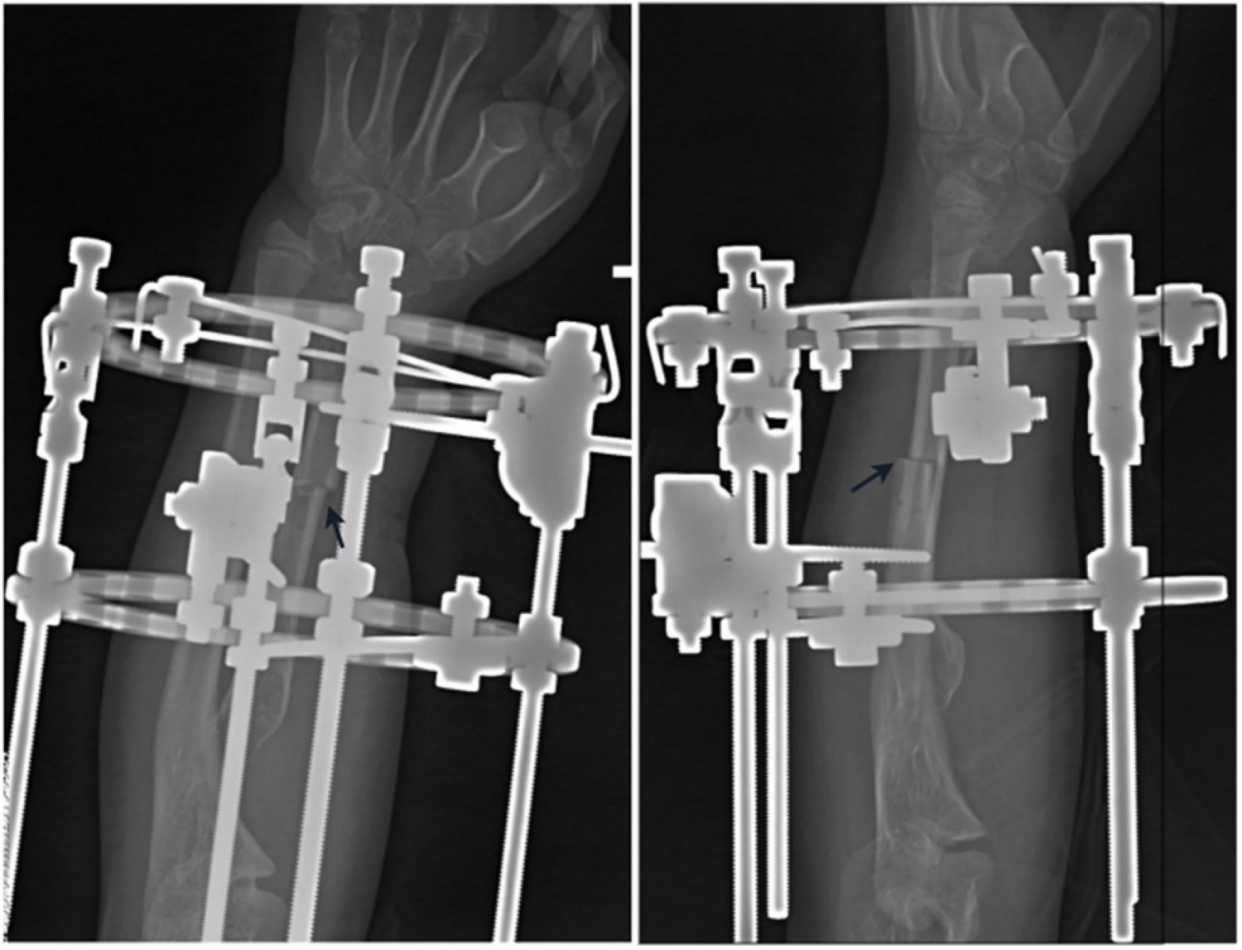
Immediate anteroposterior and lateral radiographs following radial osteotomy. The osteotomy gap(black arrow) and the fixator position are visible.

We inserted 2–4 crossed Kirschner wires or Shanz screws at both the proximal and distal ends of the planned osteotomy plane, securing them to the pre-assembled circular external fixator. Adjustable extension rods were then attached to the fixator on both the coronal plane (radial and ulnar sides) and the sagittal plane (palmar and dorsal sides) (Figure 2). A 1 cm incision was made on the radial side of the forearm to expose the radius subperiosteally. The osteotomy of the radius was performed using a microsagittal saw while maintaining the integrity of the periosteum (Figure 3).

#### 2.2.3 Postoperative adjustment protocol

Correction began on the seventh day after surgery. The daily adjustment plan included a 1.0 mm extension on the radial side, a 1.5 mm extension on the ulnar side, a 1.0 mm extension on the dorsal side, and a 1.5 mm extension on the palmar side. Geometric calculations showed that this regimen resulted in an approximate daily correction of 2° for both the ulnar inclination angle and the volar tilt angle. The lengthening speed and direction were adjusted based on intraoperative fluoroscopy and clinical feedback.

### 2.3 Postoperative management

The duration required for lengthening is calculated by comparing the measured length of the radius with that of the normal side. To determine the number of days needed, the length of the mid-axis to be lengthened is divided by 1.2 mm per day. Similarly, the ulnar inclination angle and the volar tilt angle are divided by approximately 2° per day to ascertain their respective durations. Throughout the lengthening process, it is vital to perform active functional exercises for the hand, wrist, and elbow joints, beginning in the early stages and gradually increasing intensity. Daily disinfection of the pin sites is also crucial. Once complete ossification and healing are achieved at both ends, the external fixator should be removed.

### 2.4 Evaluation indicators

#### 2.4.1 Imaging parameters

Ulnar inclination angle, volar tilt angle, and radial height were measured. Two orthopedic surgeons (XP and KT) independently performed all measurements using a standardized protocol. To assess intraobserver and interobserver reliability, measurements were repeated on 10 randomly selected patients after a 2-week interval; the intraclass correlation coefficient (ICC) was >0.85 for all parameters, indicating excellent reliability.

#### 2.4.2 Functional indicators

The wrist joint's range of motion (ROM) encompasses palmar flexion, dorsiflexion, radial deviation, and ulnar deviation. Furthermore, the ROM for forearm pronation and supination is evaluated, along with hand grip strength.

#### 2.4.3 Functional scoring

We employed the Gartland-Werley wrist joint scoring system to evaluate outcomes. Although originally designed for distal radius fractures, we selected this system because it integrates objective and subjective measures and has been used in prior Madelung deformity studies. We acknowledge its limitations in the Discussion. The system classifies results as excellent (0–2 points), good (3–8 points), fair (9–20 points), and poor (more than 20 points).

#### 2.4.4 Record of complications

All intraoperative and postoperative complications were recorded prospectively and classified by severity. Complications included pin tract infections (graded using the Checketts-Otterburn classification), nerve or vascular injuries, tendon adhesions, delayed union, nonunion, and other adverse events. Management for each complication was documented, and outcomes are reported in the Results section.

### 2.5 Statistical analysis

We used SPSS Statistics 25.0 (IBM, USA) for data analysis. We first applied the Kolmogorov–Smirnov test to confirm that the wrist range of motion, grip strength, and imaging parameters followed a normal distribution (*P* > 0.05). Measurement data are presented as mean ± standard deviation (*x* ± s). To compare preoperative and postoperative results, we conducted paired *t*-tests. Effect sizes (Cohen's d) were calculated for primary outcomes. Categorical data are reported as frequency (percentage), and the significance level *α* set at 0.05. Because the sample size was small, statistical power is limited; we address this limitation in the Discussion.

## 3 Results

**Figure 4:**
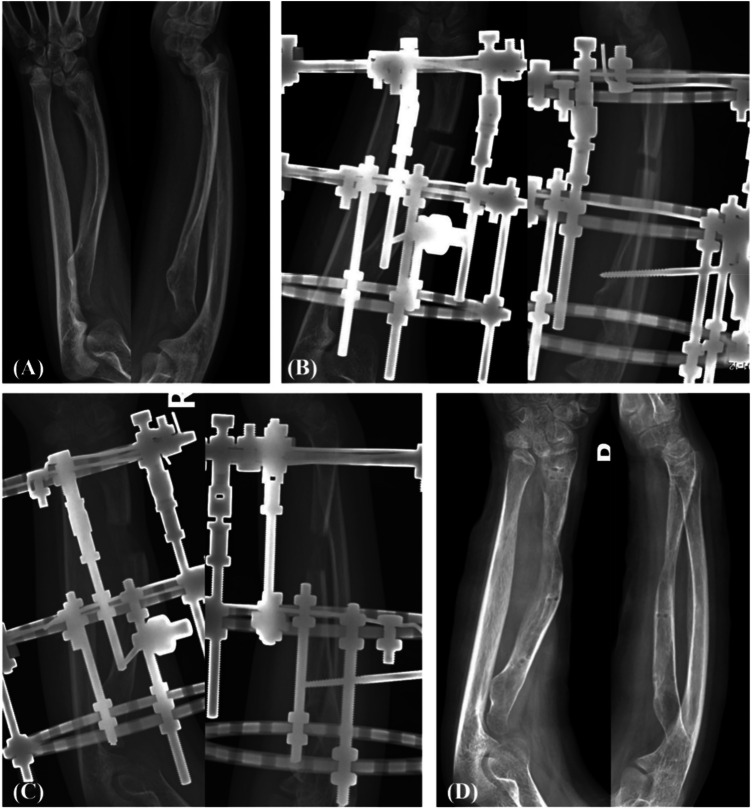
A 13-year-old female patient with right Madelung deformity. **(A)** Preoperative anteroposterior and lateral x-rays. **(B)** Thirteen days after Ilizarov application, lengthening was in progress. **(C)** Two months postoperation, lengthening was complete and ossification at the osteotomy site was visible. **(D)** Five months postoperation, full ossification was achieved and the external fixator was removed. At 4-year clinical follow-up, the patient maintained full, painless wrist function with no recurrence of the deformity.

Nineteen patients were monitored over an average of 29.2 ± 17.5 months (range 6–54 months). At the final follow-up, radiographs confirmed bony union at the osteotomy sites in all patients, and the lengthened segments showed good mineralization. Imaging data revealed significant corrections in the ulnar inclination angle, volar tilt angle of the distal radius, and radial height post-surgery, aligning closely with normal reference ranges. These improvements were statistically significant compared to pre-surgery measurements (*P* < 0.01) (Table 1). Figure 4 illustrates a representative case from our series. High-quality sequential images demonstrate progressive correction and complete bony union by five months. Long-term clinical follow-up of this patient at four years post-surgery confirmed maintained functional improvement, with a full range of motion and no pain. Postoperative assessments indicated significant enhancements in wrist joint motion, including palmar flexion, dorsiflexion, radial deviation, and ulnar deviation, as well as forearm pronation, supination, and grip strength, with most metrics returning to the normal adolescent range. These improvements were also statistically significant compared to pre-surgery levels (*P* < 0.01) (Table 2). According to the Gartland-Werley wrist joint score, 11 cases were rated as excellent, 6 as good, and 2 as fair, resulting in an excellent-good rate of 89.5%. Mild pin-tract infections occurred in 2 cases (10.5%), which resolved with oral antibiotics and enhanced local care. No severe complications, such as nerve or vascular injury, tendon adhesion, or delayed union at the osteotomy site, were observed.

**Table 1:** Comparison of preoperative and postoperative imaging parameters (*x* ± s) with effect sizes.

Indicators	Preoperative	Postoperative	*P*-value	Cohen's d
Ulnar inclination angle (°)	40.3 ± 6.1	17.7 ± 3.8	<0.01	4.2
Volar tilt angle (°)	29.8 ± 7.9	13.9 ± 5.5	<0.01	2.8
Radial height (mm)	13.5 ± 0.8	18.1 ± 0.7	<0.01	5.9

The normal reference range for ulnar inclination is 15°–25°, for volar tilt is 10°–15°, and for radial height is 20 mm–25 mm.

**Table 2:** Compares wrist joint function and muscle strength before and after surgery(*x* ± s) with effect sizes.

Indicators	Preoperative	Postoperative	*P*-value	Cohen's *d*
Palmar flexion (°)	36.1 ± 3.8	49.9 ± 3.7	<0.01	3.6
Dorsiflexion (°)	40.5 ± 3.6	51.3 ± 3.5	<0.01	3.0
Radial deviation (°)	15.5 ± 2.2	23.4 ± 3.0	<0.01	3.4
Ulnar deviation (°)	20.4 ± 2.3	31.9 ± 3.2	<0.01	4.5
Forearm pronation (°)	54.1 ± 2.9	74.4 ± 4.0	<0.01	5.3
Forearm supination (°)	54.4 ± 3.6	74.4 ± 4.2	<0.01	4.8
Hand grip strength (kg)	18.5 ± 2.7	25.4 ± 3.1	<0.01	2.2

Reference ranges for normal adolescents: palmar flexion 50°–60°, dorsiflexion 50°–60°, radial deviation 20°–30°, ulnar deviation 30°–40°, forearm pronation/supination 80°–90°. Grip strength: females 20–30 kg, males 30–40 kg.

## 4 Discussion

The results of this study showed that using the Ilizarov circular external fixator technique for progressive three-dimensional orthopedic correction effectively addressed the three main pathological changes in Madelung deformity: excessive ulnar inclination angle, volar tilt angle of the distal radius, and radial shortening. As a result, this method significantly improved wrist joint function.

Addressing Madelung deformity presents significant challenges due to its complex three-dimensional characteristics. Traditional surgical methods often fail to fully correct these complexities. The standard approach, involving radial osteotomy and internal fixation, offers only a “one-time” correction. This method necessitates a pre-operative evaluation of the radial deformity using x-ray films. The procedure involves performing an osteotomy and orthopedic correction based on the assessed ulnar inclination and volar tilt angles, followed by stabilization with a metal plate (6–9). Although this technique effectively corrects the deformity angle and facilitates direct healing at the osteotomy site, thereby shortening fracture healing time, it does not simultaneously address issues such as relative ulnar overgrowth and radial shortening. Consequently, it may leave unresolved or even exacerbate ulnocarpal impingement due to the unaddressed relative ulnar overgrowth.

In recent years, the Ilizarov technique has emerged as a recognized alternative due to its advantages in gradual lengthening and three-dimensional adjustment (10–12). This method effectively addresses radial shortening while correcting angular deformities of the radius, thereby preventing ulnocarpal impingement. Unlike traditional radial wedge osteotomy and internal fixation, the Ilizarov progressive correction technique used in this study precisely controlled daily lengthening rates on different sides. This approach significantly improved ulnar inclination and volar tilt (both *P* < 0.01) and simultaneously restored radial height (*P* < 0.01), achieving a more comprehensive anatomical reconstruction of the wrist joint. Furthermore, it avoids the risk of excessive soft-tissue stretching associated with traditional methods and allows for adjustments based on clinical responses during the lengthening process, enabling truly individualized dynamic correction.

An important factor in Madelung deformity is the Vickers ligament, an anomalous volar ligament that tethers the lunate to the radius and restricts growth. Recent studies emphasize that surgical exploration and ligament release can improve correction (4, 9). However, all patients in this cohort were older than 12 years and had physes that were near closure or fully closed, so Vickers ligament release was not performed in any case.

In this study, the average postoperative increase in radial height was 4.6 mm, which improved the biomechanical alignment of the wrist joint and created essential conditions for addressing ulnocarpal impingement. Our excellent and good outcome rate of 89.5%, coupled with a low complication rate, is consistent with recent findings in orthopedic procedures using circular external fixators or combined with three-dimensional planning (13, 14). For example, Baydar et al. reported favorable functional outcomes with the use of a circular external fixator for three-dimensional correction (3). By employing relatively straightforward operations—primarily relying on x-ray plain film planning and intraoperative adjustments—this study also achieved precise correction. This approach provides a feasible solution for performing such surgeries in centers lacking advanced three-dimensional navigation equipment.

Successful Ilizarov orthopedic procedures depend on meticulous preoperative planning, precise osteotomy, and strong patient compliance. Three-dimensional reconstruction technology, which uses x-ray and CT data, has become the gold standard for preoperative planning. This approach provides detailed insights into bone structure and assists surgeons in accurately designing the osteotomy plane before surgery, enhancing precision and effectiveness (14). Although computer-based three-dimensional simulation was not routinely employed in this study, its potential to improve osteotomy positioning and correction planning is undeniable (13, 14). In osteotomy surgery, the progressive lengthening technique is particularly effective when gradual bone length adjustment is necessary. Controlling daily lengthening to within 1.5 mm is crucial to prevent excessive traction and soft tissue injury. This method reduces postoperative complications and facilitates the gradual adaptation of soft tissues. Additionally, early and continuous functional exercise post-surgery is vital for preventing joint stiffness and promoting soft tissue adaptation, which is essential for optimal functional recovery. Studies indicate that early postoperative activity aids in restoring joint flexibility and reduces the incidence of postoperative stiffness and pain. Thus, throughout the osteotomy surgery process, from precise preoperative planning to postoperative rehabilitation exercises, each step must be carefully designed and executed to ensure optimal treatment outcomes.

Limitations: This study had several limitations. First, it was a single-center retrospective case series (Level IV evidence) with a small sample size (*n* = 19), which limited statistical power and generalizability. Second, follow-up duration varied widely (6–54 months). For example, radiographic follow-up for the patient in Figure 4 was five months after frame removal, but we documented excellent functional outcomes over a four-year clinical follow-up. This discrepancy illustrates a common challenge in retrospective studies of adolescent cohorts: obtaining long-term radiographic data from asymptomatic patients. Despite this limitation, the durable functional results provided supportive evidence for the technique's efficacy. Third, we did not include patient-reported outcome measures (PROMs) such as the DASH score, which would have provided a more comprehensive assessment of functional improvement from the patient's perspective. Fourth, the Gartland–Werley score, though applied, is not specifically validated for Madelung deformity. Fifth, no control group was available for direct comparison with traditional techniques; therefore, we could not claim superiority. Finally, the absence of blinding in radiographic measurements could have introduced bias, although we attempted to mitigate this by using two independent observers who demonstrated high reliability.

Despite these limitations, our study demonstrated that the Ilizarov technique was a feasible and safe option for correcting moderate to severe Madelung deformity in patients aged 12 years or older, particularly when radial shortening was prominent. Future research should focus on prospective multicenter studies with larger cohorts, standardized PROMs, and extended follow-up to confirm long-term benefits and to compare outcomes with alternative surgical methods.

## 5 Conclusions

The Ilizarov circular external fixator technique effectively corrects angular deformity and radial shortening in Madelung deformity in patients aged 12 years or older through progressive three-dimensional adjustments, and it significantly enhanced wrist joint function with minimal complications. For patients with moderate to severe Madelung deformity, particularly those with pronounced radial shortening and closing or closed physes, this technique is recommended. However, because of the study's limitations, these findings should be considered preliminary, and further long-term investigations are warranted.

## Data Availability

The original contributions presented in the study are included in the article/Supplementary Material, further inquiries can be directed to the corresponding author.
